# Protease‐activated receptor 2 activates CRAC‐mediated Ca^2+^ influx to cause prostate smooth muscle contraction

**DOI:** 10.1096/fba.2018-00024

**Published:** 2019-02-19

**Authors:** Madhumita Paul, Stephen F. Murphy, Christel Hall, Anthony J. Schaeffer, Praveen Thumbikat

**Affiliations:** ^1^ Department of Urology, Feinberg School of Medicine Northwestern University Chicago Illinois

**Keywords:** calcium channels, G‐protein coupled receptor, PAR2, phospholipase, prostate, smooth muscle

## Abstract

Protease‐activated receptor 2 (PAR2) is a G‐protein‐coupled receptor that contributes to prostate fibrosis and lower urinary tract symptoms (LUTS). In addition to fibrosis, aberrant smooth muscle tone in the prostate has been hypothesized to play a role. We therefore examined PAR2 expression in primary human prostate smooth muscle cells (PSMC) and studied the downstream signaling effects of PAR2 activation. Signaling pathways involved in the process were assessed using the PAR2 activating peptide SLIGKV‐NH2. We show that PAR2 is expressed in PSMC and that PAR2 activation mediates a biphasic elevation in intracellular Ca^2+^ and phosphorylation of myosin light chain 20 (MLC20), causing cellular contraction as assessed in a gel contraction assay. Intracellular Ca^2+^ flux was inhibited by a phosphoinositide hydrolysis inhibitor, U73122, showing a requirement for phospholipase C β (PLCβ) activation. PSMC expressed mRNA for L‐type voltage dependent Ca^2+^ channels (VDCC) as well as Ca^2+^ release activated channels (CRAC), a hitherto unreported finding. Secondary intracellular Ca^2+^ oscillations were abrogated only by BTP2, the CRAC channel inhibitor, but not by nifedipine, an inhibitor of VDCC. These data suggest that, PAR2 activation and subsequent Ca^2+^ entry through CRAC channels are important mechanisms in prostate smooth muscle contraction.

**Abbreviations**
BTP2: [N‐{4‐[3,5‐bis(Trifluoromethyl)‐1H‐pyrazol‐1‐yl]phenyl}‐4‐methyl‐1,2,3‐thiadiazole‐5‐carboxamide]
CRAC: Ca^2+^ release activated channels
LUTS: lower urinary tract symptoms
MLC20: myosin light chain 20
MLCK: myosin light chain kinase
PAR2: Protease‐activated receptor 2
PLCβ: phospholipase C β
PSMC: prostate smooth muscle cells
VDCC: L‐type voltage dependent Ca^2+^ channels

## 1 INTRODUCTION

PAR2 belongs to a unique family of ligand‐activated, seven transmembrane domain containing, G‐protein coupled receptors.^1^ Several tissues express the PAR2 mRNA and physiological activators of this receptor include trypsin and the mast cell‐derived serine protease, tryptase. These proteases cleave the extracellular N‐terminal domain of PAR2 and lead to a tethered ligand that initiates downstream signaling.^2^, ^3^, ^4^ The proteases that activate PAR2 are generated in diseases that involve pain, fibrosis, and tissue inflammation.^5^ In the colon of patients with irritable bowel syndrome (IBS), increased expression of mast cell tryptase and pain related neuropeptides, such as calcitonin gene related peptide (CGRP), substance P, and vasoactive intestinal peptide (VIP), were positively correlated with increased abdominal pain in these patients.^6^ Previously, we have shown in an animal model of chronic prostatitis/chronic pelvic pain syndrome (CP/CPPS), that PAR2 is involved in the pathology of fibrosis and lower urinary tract symptoms (LUTS).^7^ In patients with CP/CPPS, ultrasound evaluation of the bladder neck and posterior urethra show lesions in the fibromuscular stroma that could lead to painful contracture and alter urinary flow.^8^ Incidentally, PAR2 has been shown to play a role in the physiology of smooth muscles in different tissues.^1^, ^9^, ^10^ Although, these studies suggest a potential role for PAR2 in the involvement of prostatic smooth muscles in the pathology of urinary dysfunction, to date there is no report investigating this phenomenon.

The mechanism of smooth muscle contraction and the importance of Ca^2+^ in this process, is well defined. G protein‐mediated activation of PLC β and generation of secondary messengers IP3, cause Ca^2+^ release from the sarcoplasmic reticulum (SR) in the smooth muscle cells.^11^ The increase in cytosolic free Ca^2+^ triggers calmodulin‐dependent activation of myosin light chain kinase (MLCK), subsequently phosphorylating MLC20 to form actin myosin crossbridges and leading to muscle contraction.^12^, ^13^ Another mechanism of Ca^2+^ entry into smooth muscle cells is through VDCC and CRAC, both of which are activated by depletion of Ca^2+^ from intracellular stores.^14^ In airway smooth muscle cells, CRAC have been shown to be a major determinant of Ca^2+^ influx in these cells.^15^ In the prostate, however, the relative expression of the different CRAC proteins is not very well characterized. Also, it is not known whether Ca^2+^ influx via CRAC have any role in the contraction of smooth muscles in the prostate.

Therefore, we assessed the role of PAR2 and CRAC‐mediated Ca^2+^ entry in prostatic smooth muscle contraction. We determined the type of stromal cells in the prostate that express PAR2 and investigated the signaling pathways that are activated by PAR2 in these cells. Furthermore, we identified the various types of Ca^2+^ channels that are expressed by smooth muscle cells of the human prostate and their contribution to smooth muscle contraction. Our studies reveal a novel role of PAR2 in mediating contraction of prostatic smooth muscle through PLCβ that stimulates Ca^2+^ release, initially from internal stores and then through surface CRAC channels.

## 2 MATERIALS AND METHODS

### 2.1 Cell culture

PSMC were purchased from Lonza (Walkersville, MD) and grown in smooth muscle cells growth medium containing human epidermal growth factor (hEGF), insulin, human fibroblast growth factor‐B (hFGF‐B), and fetal bovine serum (FBS). All reagents and supplements for cell culture were obtained from Lonza.

### 2.2 Immunohistochemistry

Whole prostate from mice were excised, fixed in 10% formalin, embedded in paraffin, and were cut into 5 µm sections that were mounted on glass slides. The tissue sections were de‐paraffinized and rehydrated in xylene (VWR) followed by increasing dilutions of ethanol (Fisher). After this the tissues were boiled in sodium citrate buffer (pH 6.0) for 20 minutes to retrieve antigenic epitopes in the paraffin embedded tissues. Following epitope retrieval, tissue sections were rinsed with PBS and then blocked with 10% normal donkey serum for 30 minutes at room temperature. Slides were then washed in TBS and incubated in the following primary antibodies overnight at 4°C: mouse anti human PAR2 (clone SAM11; catalog # MABF243; EMD Millipore), rabbit anti human smooth muscle myosin heavy chain II (catalog # ab53219; Abcam), rabbit anti human vimentin (catalog # ab92547; Abcam). The tissues were rinsed in TBS and incubated in appropriate secondary antibody conjugated with fluorophore, for 1 hour at room temperature. The tissue sections were rinsed with TBS and coverslips were mounted on the tissues using ProLong® Gold antifade mounting medium (Invitrogen) containing DAPI.

### 2.3 Collagen hydrogel contraction assay

PSMC were grown in T75 flasks at 37°C with 5% CO_2_. At confluence (~4 × 10^6^ cells), the cells were trypsinized and resuspended in collagen solution provided with the collagen lattice contraction assay (Cell Biolabs Inc, San Diego, CA) to 2 × 10^6^ cells per milliliter of the collagen solution. Five hundred microliters of the collagen solution containing PSMC was plated into each well of a 24‐well plate and incubated at 37°C for 1 hour to allow the collagen matrix to polymerize. One milliliter of culture media was added on the gels and incubated for 2 days at 37°C with 5% CO_2_. On the day of the experiment, SLIGKV‐NH2 (80 µM), VKGILS‐NH2 (80 µM), or vehicle were added to individual gels. In experiments where inhibitors were used, the gels were pretreated with either U73122 (10 µM) for 30 minutes, BTP2 (12 µM) for 60 minutes, or nifedipine (10 µM) for 60 minutes, before addition of SLIGKV‐NH2 (80 µM) or vehicle. To initiate contraction, the gels were released with a sterile spatula and the free‐floating gels were imaged at time 0 and 10 minutes after release.

### 2.4 Ratiometric Ca^2+^ imaging

PSMC were grown at 2 × 10^4^ cells per well of a glass bottom 8‐well chamber slide (Lab‐Tek II chambered with coverglass # 1.5). Cells were loaded with 2.5 µM Fura‐2AM (Invitrogen) prepared in imaging buffer containing bovine serum albumin (1 mg/mL; Sigma) and incubated in the dark for 30 minutes at 37°C. Following this, the cells were rinsed once with imaging buffer to wash excess dye and incubated in fresh imaging buffer for 10 minutes at 37°C. In the experiments where inhibitors were used, cells loaded with Fura‐2AM were incubated with U73122 (10 µM), BAPTA‐AM (10 µM), BTP2 (12 µM), or DMSO for 30 minutes before commencing Ca^2+^ imaging. Ratiometric readings at 340 and 380 nm were recorded for 60 seconds to obtain baseline levels of intracellular free Ca^2+^. After this, cells were stimulated with various peptides or vehicle and readings were recorded for an additional 600 seconds. At the end of the recording period, cells were treated with ionomycin (2 µM) to produce a large Ca^2+^ response, which was used to ensure that the cells were alive during imaging and verify instrumentation. On an average, 15‐20 cells were imaged in each well with a Leica DMI6000B microscope with 40× oil immersion objective and the ratiometric images were captured using a CCD camera (Hamamatsu, Japan) and analyzed using MetaFluor Fluorescence Ratio Imaging software (Molecular Devices; LLC, San Jose, CA). All experiments were performed in triplicate. For statistical analysis, instead of pooling cells across replicates, cells were pooled within each experimental replicate and the representative line and bar graphs have been shown in the respective figures.

### 2.5 Western blotting

PSMC were lysed using RIPA lysis buffer (Thermo Scientific) containing Complete‐EDTA protease inhibitor cocktail (Roche) and phosSTOP phosphatase inhibitor (Roche) and normalized using BCA (Pierce). SDS‐PAGE was performed using the Bio‐Rad system and Criterion (Bio‐Rad) precast 4%‐12% gradient gels. Immunoblotting was performed using rabbit anti‐human myosin light chain (phospho S20) (catalog # ab2480; Abcam) and goat anti‐human GAPDH (catalog # NB300‐320; Novus Biologicals) and developed using Supersignal West chemiluminescence kit (Pierce).

### 2.6 Reverse transcriptase PCR and quantitative PCR

Total RNA was isolated from PSMC using Direct‐zol RNA MiniPrep PLUS kit (Zymo Research). For quantitative RT‐PCR, cDNA was synthesized using qScript Supermix cDNA synthesis kit (Quanta Bioscience) and quantitative PCR was performed using Sso Advanced Universal SYBR Green Supermix (Bio‐Rad), following the manufacturer's instructions, and run on the CFX Connect (Bio‐Rad) platform. The primers used to amplify the various genes are listed in Table S1 in supporting information. The threshold cycle (Ct) values of each gene was normalized with GAPDH of the same sample and data are expressed as mRNA expression relative to GAPDH. Calculations were performed in Microsoft Excel (Microsoft Corporation) and analyzed using GraphPad Prism version 7.04 for Windows (GraphPad Software).

### 2.7 Chemicals and reagents

SLIGKV‐NH2 was purchased from Abcam. VKGILS‐NH2, U73122 and nifedipine were purchased from Tocris. BTP2 was obtained from EMD millipore and BAPTA‐AM from Caymen Chemicals. SLIGKV‐NH2 and VKGILS‐NH2 were dissolved in tissue culture grade water to make stock solutions. U73122, nifedipine, BTP2, and BAPTA‐AM were dissolved in DMSO to make stock solutions which were diluted in appropriate buffers before use in experiments.

### 2.8 Statistical analysis

Quantitative data were expressed as mean ± SEM from at least three independent experiments. GraphPad Prism version 7.04 for Windows was used to analyze data for statistical significance by unpaired two tailed *t* tests and one‐way ANOVA followed by Tukey's multiple comparison test. For all statistical analyses, data were considered significantly different at *P* < 0.05.

## 3 RESULTS

### 3.1 PAR2 is expressed in the smooth muscles of the prostate

**Figure 1:**
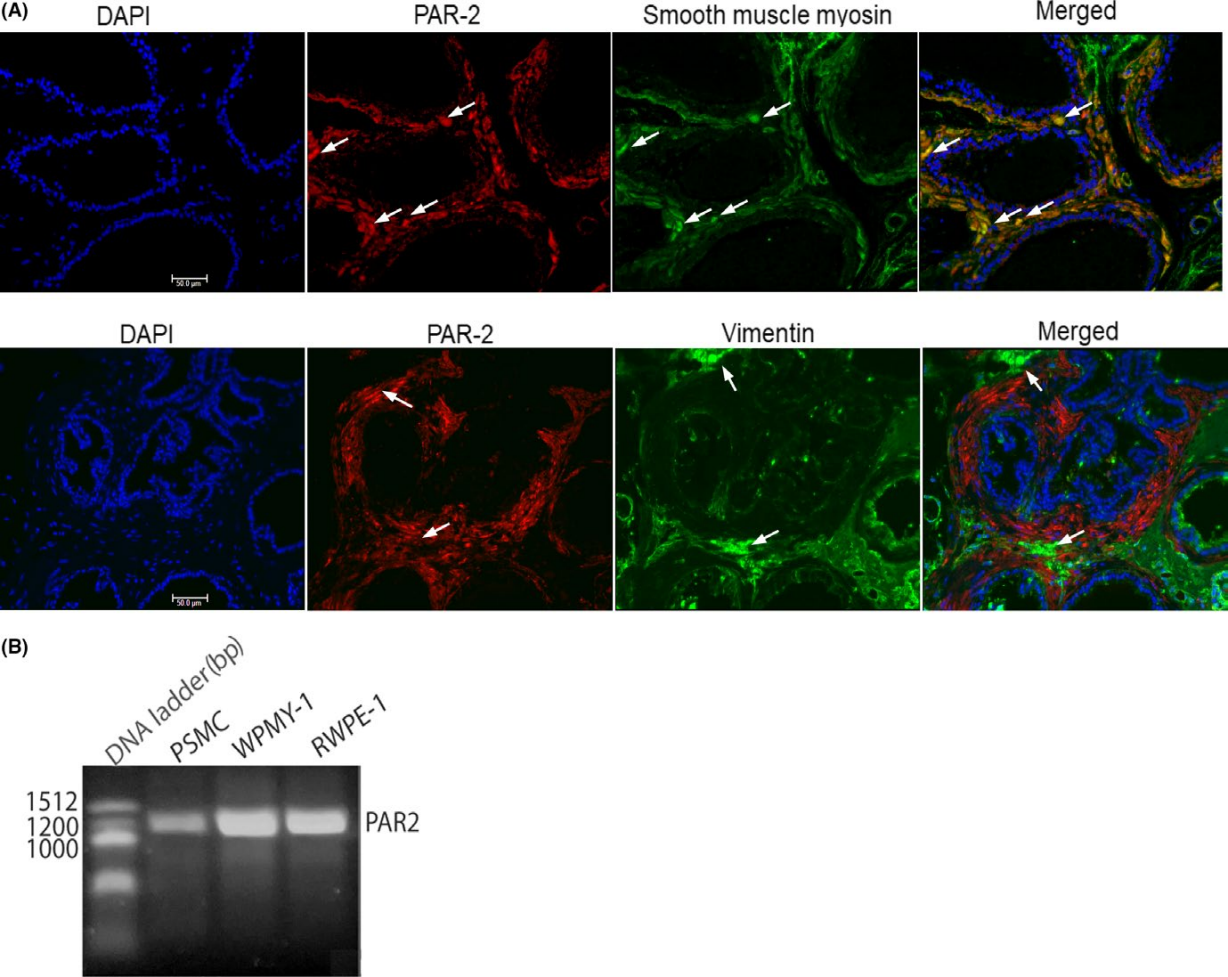
Expression of PAR2 in mice and human prostate smooth muscle cells. (A) Expression of PAR2 in smooth muscle cells in mouse prostate by immunofluorescence. Arrows in first row indicate representative cells with colocalization and in the second row cells where there si absence of colocalization and (B) in primary smooth muscle cells (PSMC) derived from human prostate by RT‐ PCR. WPMY‐1 and RWPE‐1 are myofibroblast and epithelial cell lines, respectively, derived from human prostate that are known to express PAR2 and were used as positive controls in the RT‐PCR reaction. Scale bar represents 50 µm

Previously, we have demonstrated the expression of PAR2 in the epithelial and stromal compartment of mouse prostate.^16^ In mice with experimental autoimmune prostatitis (EAP), which is a non‐infectious autoimmune driven model of murine CP/CPPS, PAR2 expression is increased more than fourfold in the prostate stroma compared to control mice.^16^ Smooth muscle cells and fibroblasts are the major constituents of the prostate stroma.^17^ Therefore, we sought to determine the stromal cell type which expresses PAR2 in the prostate. We performed immunofluorescence staining on prostate sections from mice, and reverse transcriptase PCR (RT‐PCR) to determine PAR2 expression in PSMC. Our results show that in mouse prostate, PAR2 expression colocalized mainly with cells that express smooth muscle myosin heavy chain, which is a marker for smooth muscle cells, and not with cells expressing vimentin, a marker of fibroblasts (Figure 1A). Similarly, RT‐PCR shows primary smooth muscle cells from the human prostate express PAR2 mRNA (Figure 1B). WPMY‐1 and RWPE‐1 are myofibroblast and epithelial cell lines, respectively, derived from human prostate that are known to express PAR2 and were used as positive controls in the RT‐PCR reaction.

### 3.2 PAR2 activation causes contraction of prostate smooth muscle cells

**Figure 2:**
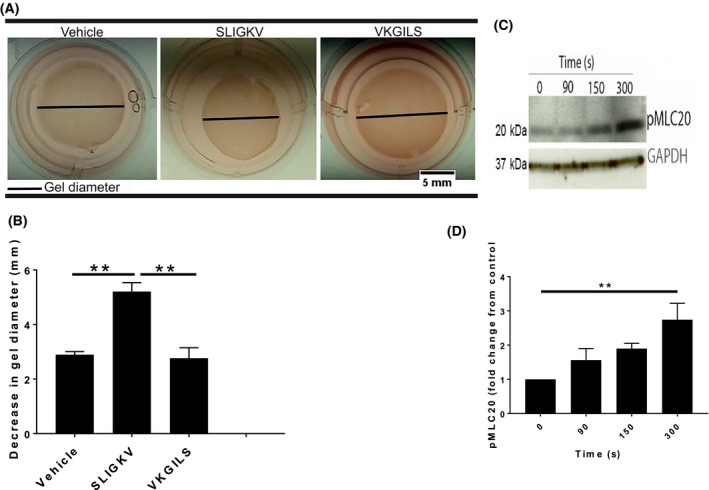
PAR2 causes contraction of smooth muscle cells from human prostate. (A and B) Decrease in diameter of collagen hydrogels after PAR2 activation with SLIGKV (80 µM). (C) Representative western blot and (D) densitometry showing time dependent increase in level of phosphorylated MLC20 in PSMC after PAR2 is activated. Data represent mean ± SEM of at least three independent experiments. Diameter of collagen hydrogels were measured in ImageJ (version 1.50i) and significance analyzed in Prism (version 7.04) with one‐way ANOVA followed by Tukey's multiple comparison test. *P < 0.05, **P < 0.01

PAR2 has been shown to cause contraction of bronchial,^10^ vascular,^1^ and esophageal smooth muscles.^9^ However, it is not known if PAR2 stimulation can cause contraction of smooth muscles in the prostate. To address the involvement of PAR2 in prostatic smooth muscle contraction, we performed an in vitro collagen hydrogel contraction assay. We treated collagen hydrogels, containing human prostate smooth muscle cells, with either SLIGKV‐NH2, a selective PAR2 agonist, VKGILS‐NH2, a control peptide, or the vehicle control which is tissue culture water. Our results show that SLIGKV‐NH2‐treated collagen hydrogels contract significantly more than the vehicle‐treated control. In comparison, the control peptide results in a contracted gel that is similar to the vehicle (Figure 2A,B). Since phosphorylated MLC20 is a prime mediator of contraction of smooth muscles, we treated PSMC with SLIGKV‐NH2 for varying lengths of time and examined phosphorylation of MLC20 using immunoblotting. Our results demonstrate that the level of phosphorylated MLC20 at 300 seconds after SLIGKV‐NH2 treatment is significantly increased in prostate smooth muscle cells (Figure 2C,D).

### 3.3 Inhibiting PLCβ reduces PAR2‐mediated contraction of prostate smooth muscle cells

**Figure 3:**
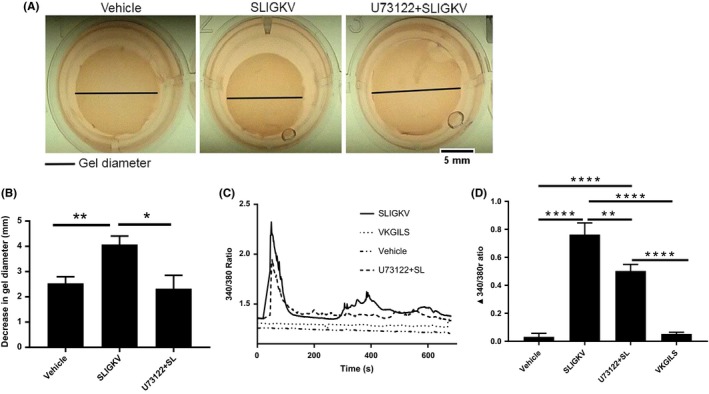
Inhibiting PLCβ prevents PAR2‐mediated contraction in PSMC and Ca^2+^ flux. (A) Representative images of collagen hydrogels and (B) bar graph demonstrating significantly reduced diameter of collagen hydrogel gels upon U73122 (10 µM) pretreatment compared to control. (C and D) Increased Ca^2+^ flux in PSMC after PAR2 stimulation with 80 µM SLIGKV (solid line), that is significantly decreased when cells are pretreated with U73122 (dashed line). Data represent mean ± SEM of three independent experiments. Diameter of collagen hydrogels were measured in ImageJ and significance analyzed by a T‐test. [Ca^2+^]_i_ was monitored using Fura‐2AM fluorescence and represented as the 340/380 nm ratio. Baseline levels of [Ca^2+^]_i_ was recorded for 60 seconds and then recorded until 600 seconds after delivery of various reagents (represented with solid arrow). Peak increase of 340/380 ratio from baseline levels after addition of reagents was analyzed in Prism (version 7.04) with one‐way ANOVA followed by Tukey's multiple comparison test. *P < 0.05, ***P < 0.001, ****P < 0.0001

PAR2 is known to mediate contraction of gastric smooth muscles through a PLCβ‐mediated signaling pathway^4^ that involves Ca^2+^ flux.^13^ PAR2 activation has been shown to elevate Ca^2+^ in many cell types.^18^, ^19^, ^20^ However, it is not known if PAR2 mediates Ca^2+^ flux in smooth muscles of the prostate. We hypothesized that PAR2 may cause PSMC contraction through PLCβ and subsequent Ca^2+^ flux. To examine this, we pretreated collagen hydrogels containing PSMC with U73122, which is a phosphoinositide hydrolysis inhibitor and does not cause increased cytotoxicity. Following pretreatment, the gels were treated with either SLIGKV‐NH2 or vehicle which is DMSO and tissue culture treated water. Our results show that U73122 pretreatment of collagen hydrogels inhibit their SLIGKV‐NH2‐mediated contraction and was no different from vehicle treated controls. In contrast, SLIGKV‐NH2 alone results in gel contraction that was significantly greater than inhibitor or vehicle‐treated gels (Figure 3A,B). This demonstrates that the activation of PAR2 mediates contraction of prostate smooth muscles via PLCβ. Next, we performed ratiometric calcium imaging in PSMC using the cell permeable Ca^2+^ indicator, Fura‐2AM. Activation of PAR2 with SLIGKV‐NH2 causes a biphasic cytosolic Ca^2+^ flux that includes an initial rapid rise in intracellular Ca^2+^ followed by sustained oscillations (Figure 3C,D). Administration of control peptide VKGILS‐NH2 or vehicle alone fail to elicit Ca^2+^ elevation in these cells (Figure 3C,D). Response to SLIGKV‐NH2 is significantly decreased when cells are pretreated with U73122, thereby confirming that PLCβ activation is a requirement for Ca^2+^ elevation in prostate smooth muscle cells (Figure 3C,D). We also confirmed that human trypsin, a known activator of PAR2, was capable of eliciting Ca^2+^ elevation in prostate smooth muscle cells (data not shown).

### 3.4 Activation of PAR2 causes Ca^2+^ flux in PSMC initially from intracellular stores and then store operated channels

**Figure 4:**
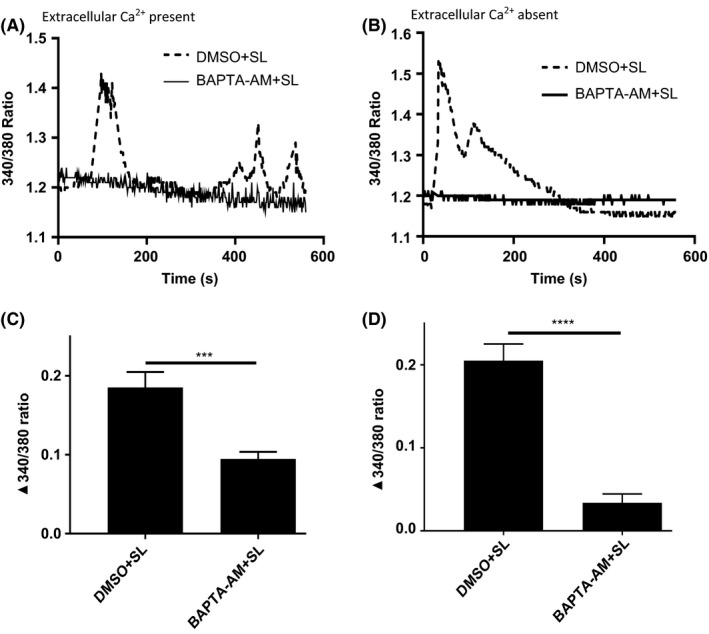
PAR2 activation causes a biphasic Ca^2+^ flux. (A and B) When Ca^2+^ is present in the external bath solution, PAR2 stimulation causes oscillatory calcium flux in PSMC (dashed line). BAPTA‐AM (10 µM) pretreatment abrogates Ca^2+^ flux with PAR2 activation (solid line). (C and D) In the absence of Ca^2+^ in the external solution, PAR2 activation causes initial Ca^2+^ flux in cells, but subsequent fluxes are abrogated (dashed line). BAPTA‐AM abolishes PAR2‐mediated Ca^2+^ flux in PSMC (solid line). Data represent mean ± SEM of three independent experiments. [Ca^2+^]_i_ was monitored using Fura‐2AM fluorescence and represented as the 340/380 nm ratio. Baseline levels of [Ca^2+^]_i_ was recorded for 60 seconds and then SLIGKV (80 µM) was delivered to cells and recorded until 600 seconds. Peak increase of 340/380 ratio from baseline levels after addition of SLIGKV was analyzed in Prism (version 7.04) with unpaired two tailed Student's *t* test. ***P < 0.001, ****P < 0.0001

Analysis of the pattern of Ca^2+ ^flux in PSMC revealed that after PAR2 stimulation there is a rapid increase in intracellular Ca^2+ ^which returns to near baseline. After this initial acute response, there are sustained oscillations in level of intracellular Ca^2+^ in PSMC (dashed line in Figure 4A). Interestingly, when PSMC are incubated in Ca^2+^‐free medium, PAR2 activation elicits only an acute Ca^2+^ flux and there is loss of subsequent oscillations (dashed line in Figure 4C). Also, chelating intracellular Ca^2+^ with BAPTA‐2AM significantly reduces PAR2‐mediated acute Ca^2+^ flux and all secondary oscillations in PSMC (Figure 4B,D), irrespective of the presence or absence of Ca^2+ ^in the extracellular buffer (solid line in Figure 4A,C). Altogether, these results suggest that PAR2 activation initially mobilizes Ca^2+^ from intracellular sources, that stimulate membrane calcium channels, allowing entry of extracellular Ca^2+^ into the smooth muscles.

### 3.5 Inhibiting Ca^2+^ release activated channels with BTP2 reduces PAR2‐mediated contraction of prostate smooth muscle cells

**Figure 5:**
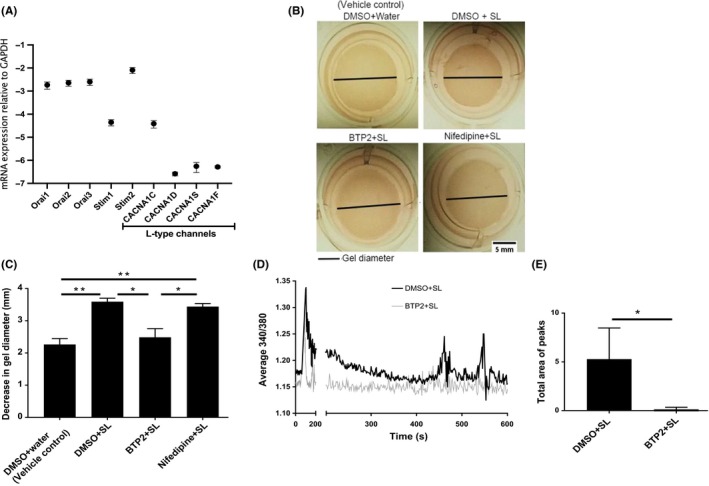
CRAC channels are involved in contraction of prostate smooth muscle cells. (A) qRT‐PCR analysis to determine expression of various CRAC and L‐type voltage channels in PSMC. (B and C) collagen hydrogels showing significantly reduced contractility upon BTP2 (12 µM) pretreatment but not after nifedipine (10 µM) pretreatment. (D and E) BTP2 (12 µM) pretreatment reduced the amplitude of the secondary Ca^2+^ oscillations in PSMC (grey line), resulting in significantly reduced area of secondary oscillations. Data represent mean ± SEM of three independent experiments. qRT‐PCR samples of respective Ca^2+^ channel mRNA relative to the expression of GAPDH as a housekeeping gene. Diameter of collagen hydrogels were measured in ImageJ (version 1.50i) and significance analyzed in Prism (version 7.04) with one‐way ANOVA followed by Tukey's multiple comparison test. [Ca2+]_i_ was monitored using Fura‐2AM fluorescence and represented as the 340/380 nm ratio. Area of the secondary oscillations was determined by area under the curve analysis performed in Prism (version 7.04) with unpaired two tailed Student's *t *test. *P < 0.05, **P < 0.01

Next, we sought to determine the specific type of membrane Ca^2+^ channels that are activated following PAR2 stimulation. Smooth muscles have been shown to express different types of voltage‐dependent Ca^2+^ channels (VDCC), such as L‐type channels, and also Ca^2+^ release activated channels (CRAC) channels, such as the Orai channels.^21^ These two types of Ca^2+^ channels have multiple isoforms that are expressed in a tissue‐specific manner.^14^ However, the expression of these various VDCC and CRAC channels has not been well characterized in smooth muscles of the human prostate. We performed quantitative RT‐PCR (qRT‐PCR) and determined the expression of L‐type channels Ca_v_1.2 (CACNA1C), Ca_v_1.3 (CACNA1D), Ca_v_1.1 (CACNA1S), and Ca_v_1.4 (CACNA1F), and CRAC channels Orai1, Orai2, Orai3, STIM1, and STIM2 in PSMC. The STIM family of proteins are Ca^2+^ sensors in the sarcoplasmic/endoplasmic reticulum that sense Ca^2+^ depletion in the SR and couple with the transmembrane Orai channels to facilitate Ca^2+^ entry. We compared the expression of the above Ca^2+^ channels in PSMC with the expression of the housekeeping gene GAPDH. Our results show that PSMC express differential levels of the various isoforms of individual Ca^2+^ channels (Figure 5A). Among the Orai channels, Orai1, Orai2, and Orai3 are equally expressed at high levels, while STIM2 has more expression than STIM1 in PSMC. As far as the L‐type channels are concerned, PSMC have maximal expression of Ca_v_1.2 followed by very low expression of Ca_v_1.1, Ca_v_1.3, and Ca_v_1.4. To identify the type of Ca^2+^ channel responsible for causing the secondary flux in intracellular Ca^2+^ and their relative contribution to PSMC contraction, we used BTP2, an inhibitor of CRAC channels, and nifedipine, an inhibitor of L‐type channels. In the in vitro gel contraction assay, we observe that while preincubation of collagen hydrogels with BTP2 significantly reduces contraction of PSMC compared to control, nifedipine is unable to do the same (Figure 5B,C). Concomitantly, BTP2 pretreatment reduces the amplitude and total area of the secondary oscillations following SLIGKV‐NH2 treatment in PSMC (Figure 5D,E). These data indicate that activation of CRAC channels after PAR2 stimulation contribute to contraction of smooth muscles in the prostate and that L‐type VDCC are not involved in this process.

## 4 DISCUSSION

**Figure 6:**
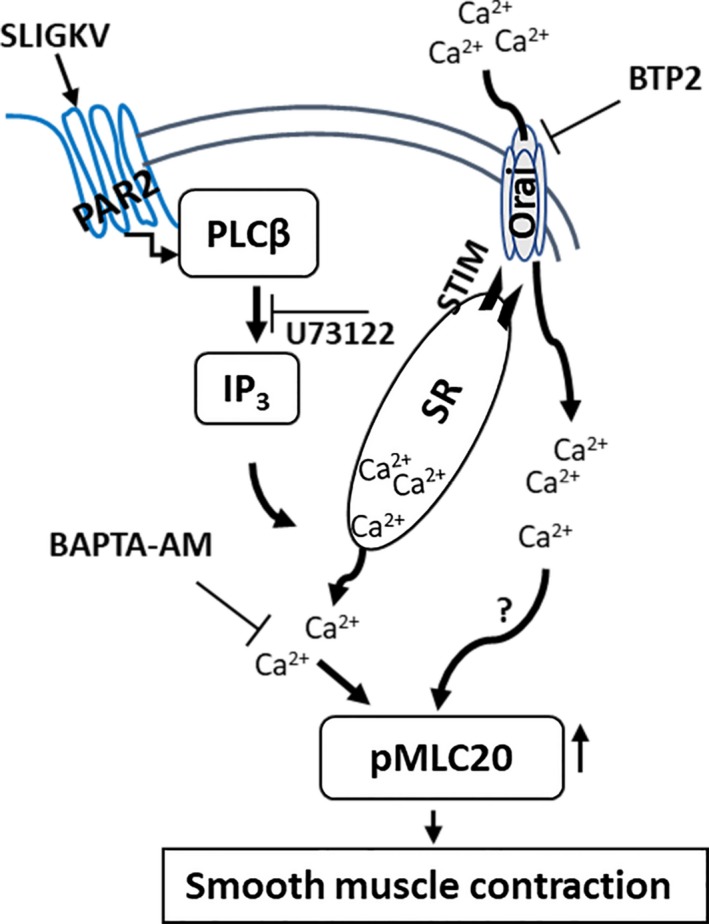
Schematic representation of signaling pathway activated by PAR2 to cause smooth muscle contraction in prostate. Stimulation of PAR2 activates PLCβ/IP_3_ signal cascade to release Ca^2+^ from sarcoplasmic reticulum (SR) in smooth muscle cells of the prostate. The released Ca^2+^ initiates smooth muscle contraction via MLC kinase activation and increased phosphorylation of MLC20. Furthermore, Ca^2+ ^released from the SR also activates STIM channels on the SR membrane, that activate the Orai CRAC channels, causing an influx of extracellular Ca^2+^ that sustains smooth muscle contraction by a mechanism that may involve MLC20 phosphorylation

The key findings of our study are that PAR2 is expressed in smooth muscles of the prostate and upon stimulation, PAR2 causes contraction of these cells by activating surface CRAC channels (Figure 6). To the best of our knowledge, this is the first report describing the involvement of PAR2 and CRAC in mechanisms mediating prostatic smooth muscle contraction.

The PAR2 gene is widely expressed in most tissues of the human body, except the brain and skeletal muscles.^2^, ^3^ We have previously shown PAR2 expression in the prostate stroma.^16^ In the present study, PAR2 immunoreactivity was localized mainly in the smooth muscle compartment of the murine prostate stroma and primary smooth muscle cells from human prostate. In an attempt to determine the function of PAR2, we sought to investigate the effect of PAR2 stimulation in prostatic smooth muscles. Sriwai et al^4^ and Ha et al^9^ showed that PAR2 activation caused biphasic contraction of gastric and esophageal smooth muscles. However, our study has further characterized the different sources of the intracellular Ca^2+^ that cause the distinctive biphasic Ca^2+^ flux and demonstrated the involvement of CRAC channels, and not L‐type VDCC, in PAR2‐mediated contraction of prostate smooth muscles. Traditionally, CRAC channels were thought to mobilize cellular Ca^2+^ signaling in many non‐excitable cells.^22^, ^23^ However, the discovery of CRAC channels in smooth muscles has triggered interest in defining the role of these channels in smooth muscle function.^14^, ^24^ Most recent studies have shown that aberrant activation of STIM1‐Orai1 impairs the control of cellular Ca^2+^ levels that cause vascular dysfunction associated with arterial hypertension.^25^ In a model of chronic hypoxia induced pulmonary hypertension, the expression of Orai1 and Orai2 were seen to be upregulated in pulmonary arteries and pulmonary arterial smooth muscle cells after chronic hypoxia exposure.^26^ Increased expression of STIM1 and Orai1 proteins have also been implicated in vascular smooth muscle cell remodeling following balloon injury in the rat carotid artery.^27^ Furthermore, this study also shows that upregulation of STIM1 and Orai1 is required for nuclear translocation and activation of the transcription factor nuclear factor for activated T cell (NFAT) to allow proliferation of the vascular smooth muscle cells. Taken together, these studies demonstrate that STIM/Orai coupling contributes to the phenotypic remodeling of vascular and pulmonary smooth muscles in response to injury. Therefore, it was argued that SOC‐mediated intracellular Ca^2+^ increase is not necessary for smooth muscle contraction.^24^, ^28^ However, this notion has been challenged in light of new evidence that showed contraction of airway smooth muscle cells in precision cut lung slices can be blocked by inhibiting STIM/Orai coupling.^29^ In contrast, blocking L‐type channels had reduced effect on contraction of these cells.^29^ These studies support our observations in prostate smooth muscle cells where depletion of intracellular stores of Ca^2+^ after PAR2 activation causes CRAC channel‐mediated Ca^2+^ flux and subsequent contraction of smooth muscles in the prostate. We have also shown that all three human Orai homologs (Orai 1, 2, and 3) and two STIM homologs (STIM1 and 2) are expressed in smooth muscle cells isolated from human prostate. This pattern of expression has also been shown in human airway smooth muscle cells.^15^ While Chen et al^29^ did not determine the specific type of Orai or STIM homolog responsible for contraction of airway smooth muscle cells, Peel et al^15^ showed that Orai1 and, potentially, Orai3 are responsible for store operated Ca^2+^ influx in airway smooth muscle cells. Our qRT‐PCR data suggest that in human prostate smooth muscle cells Orai1, 2, and 3 are equally expressed. However, further experiments have to be performed to determine which Orai channel is contributing to contraction of these cells.

PAR2 is a G‐protein coupled receptor that, as demonstrated by us in this present study and others,^4^ requires PLCβ signaling to cause smooth muscle contraction in the prostate. Activation of α‐adrenergic receptors also activate a similar signaling pathway in the prostate.^30^, ^31^ Traditionally, drugs blocking the α‐adrenergic receptor (α‐blockers) have been used to treat voiding symptoms in male patients presenting with benign prostatic hyperplasia (BPH) by inhibiting contraction of bladder neck smooth muscles.^32^, ^33^, ^34^ α‐blockers have also been used to treat urinary symptoms associated with CP/CPPS. However, this therapeutic regimen has a modest impact on relieving urinary symptoms in patients of CP/CPPS.^35^, ^36^ Previous work from our laboratory has shown that patients and animal model of CP/CPPS have elevated levels of mast cell tryptase, which is the naturally occurring activator of PAR2.^16^, ^37^ In the murine EAP model of CP/CPPS, there is also increased expression of PAR2 and these mice have associated urinary dysfunction. When PAR2 is neutralized with antibodies, it normalizes voiding frequency and bladder capacity in these CP/CPPS animals.^7^, ^16^ Therefore, based on these studies and our current findings, we think that the limited efficacy of α‐blockers in treating voiding symptoms in patients of CP/CPPS may be due to activation of non‐adrenergic receptors, such as PAR2, which can initiate smooth muscle contractions as α‐adrenergic receptors themselves. In fact, Hennenberg et al^38^ have alluded to this possibility when they showed that in human prostate tissue endothelin‐1 can induce smooth muscle contraction and induce contractile forces that have similar magnitude to those caused by noradrenaline. Similarly, there may be a likelihood that in patients of CP/CPPS with urinary symptoms, chronic activation of PAR2 by high levels of mast cell tryptase in the prostate may cause smooth muscle contractions that may be complementary to those caused by noradrenalin. This could explain why α‐blockers have had partial success in treating voiding dysfunction in CP/CPPS patients.

In summary, this study provides evidence that PAR2 can cause contraction of prostate smooth muscle cells via a pathway that is known to be activated by α‐adrenergic receptors to have a similar contractile effect on smooth muscles. Future studies are aimed at understanding the effect of simultaneous activation of PAR2 and α‐adrenergic receptor on prostate smooth muscles and exploring the possibility of using PAR2 blockade, standalone or in combination, with α‐blockers to better manage urinary functions associated with CP/CPPS.

## CONFLICT OF INTEREST

The authors declare that they have no conflicts of interest with the contents of this article.

## AUTHOR CONTRIBUTIONS

PT and MP designed research. MP performed experiments, analyzed data, and wrote the article. SFM performed experiments, and analyzed data. PT secured funding and wrote the article. CH helped in reagent procurement. AJS provided critical feedback and manuscript critique.
