# Supplementary material for: Protease‐activated receptor 2 activates CRAC‐mediated Ca2+ influx to cause prostate smooth muscle contraction
Source: FASEB Bioadv. 2019 Feb 19;1(4):255–64. doi: 10.1096/fba.2018-00024 (PMC6563600; doi:10.1096/fba.2018-00024)
Supplement: Supplementary file 1 [file FBA2-1-255-s001.docx]

Supplementary Material 1

Protease activated receptor 2 activates CRAC mediated Ca^2+^ influx to cause prostate smooth muscle contraction

Madhumita Paul, Christel Hall, Anthony J Schaeffer, Praveen Thumbikat*

Table S1: List of primers used in RT-PCR and qRT-PCR reactions

| Gene | Forward primer | Reverse primer |
| --- | --- | --- |
| PAR2 | 5’-ATGCGGAGCCCCAGCG-3’ | 5’- ATAGGAGGTCTTAACAGTGGTTGAA-3’ |
| ORAI1 | 5’- GCTTCGCCATGGTGGCAAT-3’ | 5’- GGGACTCCTTGACCGAGTTG-3’ |
| ORAI2 | 5’- AACGTCCCAGGGATGGAAGT-3’ | 5’- AGCCAGGCAGGTCATTTATACG-3’ |
| ORAI3 | 5’- GGCTACCTGGACCTCATGG-3’ | 5’- ATGGCCACCATGGCGAAG-3’ |
| STIM1 | 5’- TTGTCCATGCAGTCCCCTA-3’ | 5’- ATTCGCCGCAGAGATACCC-3’ |
| STIM2 | 5’- AGGATAGCAGTGCACGAACC-3’ | 5’- AGTTATGAGGTGGGCGTGTT-3’ |
| CACNA1C | 5’- GATCCTAGGCAATGCAGACT-3’ | 5’- TGATTGCACTGGACTGGATG-3’ |
| CACNA1D | 5’- CGGCTCCTACCTCTTGGTGA-3’ | 5’- AGTTTGCCTCGTTCGCGT-3’ |
| CACNA1S | 5’- CTCCAGCGGGGGACTGTATT-3’ | 5’- ACGTAGGGTAGGGCCTGGAA-3’ |
| CACNA1F | 5’- CTGGAACACGTTTGACGCTC-3’ | 5’- TCCTCAGAGCTCTCGCCAA-3’ |
